# Suppression of Virus Specific Immune Responses by IL-10 in Acute Dengue Infection

**DOI:** 10.1371/journal.pntd.0002409

**Published:** 2013-09-05

**Authors:** Gathsaurie Neelika Malavige, Chandima Jeewandara, K. M. Luckmaal Alles, Maryam Salimi, Laksiri Gomes, Achala Kamaladasa, S. D. Jayaratne, Graham Stuart Ogg

**Affiliations:** 1 Centre for Dengue Research, Faculty of Medical Sciences, University of Sri Jayawardanapura, Gangodawila, Nugegoda, Sri Lanka; 2 Medical Research Council Human Immunology Unit, Weatherall Institute of Molecular Medicine, University of Oxford, Oxford, United Kingdom; 3 Department of Medicine, Faculty of Medical Sciences, University of Sri Jayawardanapura, Gangodawila, Nugegoda, Sri Lanka; 4 Department of Dermatology, Churchill Hospital, Oxford, United Kingdom; Pediatric Dengue Vaccine Initiative, United States of America

## Abstract

**Background:**

Elevated IL-10 has been shown to be associated with severe dengue infection (DI). We proceeded to investigate the role of IL-10 in the pathogenesis of acute DI.

**Materials and methods:**

*Ex vivo* and cultured IFNγ ELISpot assays for dengue virus (DENV) NS3 protein and non dengue viral proteins were carried out in 26 patients with acute DI (16 with dengue haemorrhagic fever) and 12 healthy dengue seropositive individuals from Sri Lanka. DENV serotype specific (SS) responses were determined by using a panel of SS peptides.

**Results:**

Serum IL-10 level were significantly higher (p = 0.02) in those who did not have *in vitro* responses to DENV-SS peptides (mean 144.2 pg/ml) when compared to those who responded (mean 75.7 pg/ml). DENV-NS3 specific *ex vivo* IFNγ ELISpot responses were also significantly lower (p = 0.0001) in those who did not respond to DENV-SS peptides (mean 42 SFU/million PBMCs) when compared to those who responded to DENV-SS peptides (mean 1024 SFU/million PBMCs). Serum IL-10 levels correlated significantly (p = 0.03) and inversely (Spearmans R = −0.45) with *ex vivo* DENV-NS3 specific responses but not with *ex vivo* non DENV specific responses (Spearmans R = −014, p = 0.52). Blockage of IL-10 *in vitro* significantly increased (p = 0.04) the *ex vivo* IFNγ ELISpot DENV-NS3 specific responses but had no effect on responses to non DENV proteins.

**Conclusion:**

IL-10 appears to contribute to the pathogenesis of acute dengue infections by inhibiting DENV-specific T cell responses, which can be restored by blocking IL-10.

## Author Summary

Dengue viral infections have become one of the most important mosquito borne viral infections in the world and are one of the major emerging infectious diseases. The occurrence of dengue haemorrhagic fever is thought to result from a complex interplay between the virus, host genetics and host immune factors. Patients with more severe clinical disease have been shown to have higher serum IL-10 levels and IL-10 has also been shown to be associated with poorer disease outcome in other viral infections. Therefore, in this study, we proceeded to investigate the role of IL-10 in the pathogenesis of dengue infections and its effect on DENV-specific T cells. We found that both DENV- serotype-specific (SS) specific T cell responses and DENV-NS3 specific responses were impaired in patients with higher serum IL-10 levels. Serum IL-10 levels did not appear to have any effect on non dengue viral protein specific responses. IL-10 blockade significantly increased IFNγ production, in response to DENV-NS3 peptides but not to non dengue viral proteins in acute dengue infection. Therefore, our results suggest that IL-10 could be contributing to disease pathogenesis by inhibiting DENV-specific T cell responses.

## Introduction

Dengue viral infections have become one of the most important mosquito borne viral infections in the world and are one of the major emerging infectious diseases. Dengue is a major public health problem in over 100 countries in the tropical and sub-tropical regions. It has been predicted that 390 million dengue infections occur per year resulting in approximately 96 million clinically apparent infections [1]. Although the case fatalities due to dengue viral infection are around 1–5% depending on the country and epidemic [2], infection is thought to be fatal in approximately 2.5% individuals with dengue haemarhagic fever (DHF) [3]. Currently there are no effective antivirals to treat this infection and there is no effective vaccine.

Infection with the dengue virus (DENV), may manifest as asymptomatic or a mild febrile illness, DF, DHF or dengue shock syndrome (DSS), which can be fatal [3], [4]. Dengue may occur due to infection with any one of the four DENV serotypes. Initial infection with a particular serotype is known as a primary infection, which is usually asymptomatic or results in mild disease manifestations [5]. Primary infection can occasionally associate with severe disease, but it is subsequent infection with other serotypes (secondary dengue infections) that more commonly leads to severe disease [5]. Severe clinical disease manifestations such as DHF/DSS are thought to result from a complex interplay between the virus, host genetic background and host immune factors. However, many questions regarding factors that lead to severe disease and the pathophysiology of dengue viral infection itself remain unanswered. Both cross reactive antibodies and T cells to the previous infecting DENV serotype are thought to contribute to disease pathogenesis [5], [6].

Highly cross reactive T cells have been shown to occur in patients with acute dengue viral infection [7]–[9], and are thought to contribute to disease pathogenesis as they are believed to be suboptimal in clearing the virus [10]. Some have expressed different opinions regarding the possibility of cross reactive T cells contributing to disease pathogenesis [11]. In fact a more recent study showed that a large proportion of individuals living in dengue endemic areas had a high magnitude of polyfunctional responses to multiple CD8+ T cell epitopes of the DENV, which were associated with protection [12]. Therefore, it is possible that rather than causing immunopathology, DENV-specific T cells responses may actually be protective in acute dengue infection. Massive apoptosis of T cells has been shown to occur in acute dengue viral infection [13], [14]. Furthermore, it has been shown that patients with more severe forms of disease have higher viral loads and prolonged viraemia [15], [16]. Therefore, it is possible that an impaired T cell response in acute dengue is associated with delayed viral clearance leading to clinical disease severity.

We previously found that serum IL-10 levels were associated with T cell apoptosis although in subsequent *in vitro* experiments we found that IL-10 itself did not cause apotosis of T cells in PBMCs in healthy volunteers [13]. In these experiments, PBMCs of healthy individuals were incubated with varying concentrations of human recombinant IL-10 for 24 hours and we found that in these *in vitro* experiments higher IL-10 levels were not associated with apoptosis of T cells in these healthy individuals. However, these experiments do not rule out IL-10 causing T cell apoptosis in patients with acute dengue infection as we did find that serum IL-10 levels correlated well with T cell apoptosis in patients with acute dengue. Patients with more severe clinical disease have been shown to have higher serum IL-10 levels [17], [18] and IL-10 has also been shown to be associated with poorer disease outcome in other viral infections [19], [20]. Therefore, in this study, we proceeded to investigate the role of IL-10 in the pathogenesis of dengue viral infections and its effect on DENV-specific T cells. We found that both DENV- serotype-specific (SS) specific T cell responses and DENV-NS3 specific responses were impaired in patients with higher serum IL-10 levels. Serum IL-10 levels did not appear to have any effect on non dengue viral protein specific responses. IL-10 blockade significantly increased IFNγ production, and other antiviral responses such as CD107a expression and TNFα production in response to DENV-NS3 peptides but not to non dengue viral proteins in acute dengue infection.

## Materials and Methods

### Patients

26 adult patients (mean age 26.3, SD±7.2) with clinical features suggestive of acute dengue viral infection and confirmed to be antibody positive (see below) were admitted to a general medical ward in a tertiary care hospital in Colombo and were enrolled in the study following informed written consent. Blood samples were collected during day 4–5 of illness (day 1 was considered as the first day of fever) in all patients and during the convalescent phase in 12 patients (10–14 days from collection of first blood sample and approximately one week after the patients had left the hospital). Blood was also collected from 12 healthy dengue seropositive individuals. Serial recordings of their clinical features and laboratory investigations (platelet counts, haematocrits, white cell counts) were made until they were discharged from the hospital in order to determine the severity of dengue infection. The severity of acute dengue infection was classified according to the 2011 WHO guidelines [3]. Patients with DHF with a pulse pressure of ≤20 mmHg were classified as having shock [3]. Of the 26 patients, 16 patients had DHF based on the 2011 WHO criteria and 10 patients had DF. Of the 16 patients with DHF, 6 patients developed DSS as their pulse pressure dropped to 20 mmHg during the course of the illness.

The study was approved by the Ethical Review Committee of the University of Sri Jayawardanapura. Informed written consent was obtained from all subjects who participated in the study.

### Peptides

A panel of 17 peptides which were previously described and found to be serotype specific (SS) originating from highly conserved regions of the four DENVs was used [21]. These were 20mer peptides which were synthesized in house in an automated synthesizer using F-MOC chemistry. The purity of the peptides was determined to be greater than 90% by high-pressure liquid chromatography analysis and mass spectrometry. There were four peptides specific to DEN-1, five specific to DEN-2, four specific for DEN-3 and four specific for DEN-4. The DENV- NS3 peptides were 20 mer peptides overlapping by 10 amino acids, which spanned the whole length of the DENV-3 NS3 protein. The synthetic NS3 20mer peptides were pooled together to represent the whole NS3 protein. The FEC peptides that were used contained a panel of 23, 8–11 amino acid CD8+ T cell epitopes of Epstein Barr virus (EBV), Flu and CMV viruses and have been used as quality control in ELISpot assays [22].

### Ex vivo ELISpot assays

*Ex vivo* Elispot assays were performed as previously described [23], [24] in 26 patients with acute dengue infection and the healthy volunteers. ELISpot plates (Millipore Corp., Bedford, Massachusetts, USA) were coated with anti-human IFNγ antibody overnight (Mabtech AB, Nacka, Sweden). For *ex vivo* ELISpot assays, 0.1×10^6^ PBMC were added to a final volume of 200 µl. DENV-NS3 overlapping peptides and FEC peptides were added at a final concentration of 10 µM as previously described [10], [25]. All peptides were tested in duplicate. PHA was always included as a positive control and media alone with the PBMCs was included as a negative control. The plates were incubated overnight at 37°C and 5% CO_2_. The cells were removed and the plates developed with a second biotinylated Ab to human IFNγ and washed a further six times. The plates were developed with streptavidin-alkaline phosphatase (Mabtech AB) and colorimetric substrate, and the spots enumerated using an automated ELISpot reader. Background (cells plus media) was subtracted and data expressed as number of spot-forming units (SFU) per 10^6^ PBMC. *Ex vivo* ELISpot assays were used to determine T cell responses to DENV-SS peptides in a previous cohort of patients with acute dengue infection (n = 20). We found that the majority of patients did not have *ex vivo* responses to SS peptides during acute symptomatic infection and in the few who had responses, the frequency of responses were very poor (unpublished data). However, after acute symptoms had resolved and the overall lymphopaenia recovered *ex vivo* SS responses became detectable. In contrast, cultured T cells specific to SS peptides were detectable in patients with acute dengue infection.

### Cultured ELISpot assays

Cultured ELISpot assays were performed on all the acute samples, on 12 convalescent samples and on the healthy volunteers to detect responses to DENV-SS peptides. PBMC from each donor were incubated with a pool of peptides consisting of all the 17 SS peptides. Briefly, 5.0×10^6^ PBMCs were incubated for 10 days with 200 µl of 40 µM peptide pool in a 24 well plate. IL-2 was added on day 3 and 7 at a concentration of 100units/ml. All cell lines were routinely maintained in RPMI 1640 supplemented with 2 mM L-glutamine, 100 IU/ml penicillin and 100 µg/ml plus 10% human serum at 37°C, in 5% CO_2_. T cell lines were tested individually after 10 days culture for responses to the 17 SS peptides.

### Intracellular cytokine assays

To determine IL-10 production, *ex vivo*- PBMCs were stimulated at 1×10^6^ to 2×10^6^/ml in RPMI 1640 plus 10% FCS with DENV-NS3 overlapping peptides and PMA and ionomycin for 16 hours according to manufacturer's instructions in the presence of Brefeldin A (Biolegend, USA). For TNFα detection and CD107a expression, cells were washed and stained with anti CD3 APC (Biolegend, USA), anti CD4 PerCP (Biolegend, USA) and anti CD8 PE (Biolegend, USA). Cells were then permeabilized and fixed with Cytofix/Cytoperm (BD Biosciences) and then stained for intracellular TNFα FITC or CD107a FITC (Biolegend, USA). Propidium Iodide was used in the CD107a assays to gate out dead cells. For detection of IL-10, cells were stained with CD3 APC, CD14 (FITC, Biolegend USA), CD19 PerCP (Biolegend, USA) and then fixed and stained for intracellular IL-10 PE (Biolegend, USA). Cells were acquired on a Partec Cyflow Cube 6 and analyzed with De novo FCS Express version 4.

### Quantification of cytokines

IL-10 quantitative cytokine assays were done in duplicate on serum according to manufacturer's instructions. Serum IL-10 levels (Mabtech, Sweden), MIF levels (Biolegend, USA) and IL-21 levels (Biolegend, USA) were done in all serum samples and were also done in all the serum samples of patients with acute infection and healthy volunteers and also in serum samples obtained during the convalescent phase. Both IL-10 and TGFβ levels (Mabtech, Sweden) were done in *ex vivo* ELISpot supernatants of the unstimulated wells (containing PBMCs and media alone), DENV-NS3 stimulated wells and FEC peptide stimulated wells. All reactions were carried out in duplicate.

### Detection of DENV-NS3 specific responses and responses to non DENV peptides with IL-10 blockage

To determine the effect of IL-10 blockage on PBMCs using *ex vivo* IFNγ ELISpots, the ELISpots were coated and the PBMCs added as usual, and before the peptides were added, the PBMCs were incubated with anti IL-10 (5 µg/ml, Biolegend, USA) and anti IL-10R (10 µg/ml, Biolegend, USA) for 1 hour. After blockage for 1 hour, ELISpots were carried out as usual. To determine the effect of IL-10 blockage on TNFα production and CD107a expression, the PBMCs were incubated with anti IL-10 antibody and anti IL-10R antibody for 1 hour before peptides were added. After blockage for 1 hour, the ICS was done as usual.

### Dengue virus specific RT-PCR

Dengue virus RNA was extracted from serum using QIAmp viral RNA mini kit (Qiagen). RNA was reverse transcribed and the PCR was performed by using primer and conditions as previously described [26]. When determining the serotype of the infecting DENV, positive controls for DENV-1, DENV-2, DENV-3 and DENV-4 were used in all experiments.

### Serology

DENV infection was confirmed by testing the serum samples which were collected after day 6 of illness with a commercial capture-IgM and IgG enzyme-linked immunosorbent assay (ELISA) (Panbio, Brisbane, Australia). The ELISA was performed and the results were interpreted according to the manufacturer's instructions. Patients who only had dengue virus specific IgM were classified as having a PD infection while those who had a positive result for both IgM and IgG were classified as having a SD infection [2].

### Statistical analysis

Statistical analysis was carried out using PRISM version 6. As the data was not normally distributed, differences in means were compared using the Mann-Whitney t test (two tailed). To determine the effect of IL-10 blockade on DEN-V NS3 specific T cells, Wilcoxon matched-pairs sign ranked test (two tailed) was used. To determine positive and negative associations, the Spearman's correlation test was used (two tailed).

## Results

Table 1 shows the clinical and laboratory characteristics of patients with acute dengue infection. Although some patients with DF had thrombocytopenia (platelet counts <100,000 cells/mm^3^), they were classified as having DF as they did not fulfill the criteria to be classified as DHF based on the 2011 WHO dengue guidelines. Of the patients who were classified as having DHF, the day their illness was most severe was day 6 (SD±1.1). The lowest white cell counts and lymphocyte counts were observed approximately 12–24 hours prior to the most critical phase of the illness, while the lowest platelet counts were seen approximately 12 hours after the onset of the critical phase of the illness. The most critical time point in the illness in patients with DHF was determined by documenting the lowest pulse pressure, blood pressure, and highest haematocrit.

**Table 1:** Clinical and laboratory characteristics of patients with acute dengue infection.

Clinical feature	Dengue fever N = 10	DHF N = 16
Number of days in hospital	3.6 (SD±0.5)	4.7 (SD±1.2)
Hepatomegaly	3	6
Pleural effusions or ascites	0	5
Bleeding manifestations	0	3
Platelet counts on admission (cells/mm^3^)	101,000 (SD±22,568)	92,400 (SD±40,419)
Lowest platelet count (cells/mm^3^)	84,300 (SD±28,848)	34,933 (SD±17,190)
Lowest white cell count (cells/mm^3^)	4,060 (SD±2,363)	2,627 (SD±1,289)
Lowest lymphocyte count (cells/mm^3^)	1,579 (SD±1,027)	966.7 (SD±569)
Asparatate transaminase levels (IU)	168.5 (SD±123.6)	219.9 (SD±205.5)
Alanine transaminase levels (IU)	133.1 (SD±99.8)	146.1 (SD±156.4)

### DENV-serotype specific T cell responses

T cell responses to DENV SS peptides were determined by *in vitro* cultured ELISpot assays where PBMCs were cultured with a panel of previously defined SS peptides for each DENV serotype [21]. 10/26 patients (3/16 patients with DHF and 7/10 patients with DF) with acute dengue infections responded to at least one SS peptide of a DENV serotype, while all 12 healthy dengue seropositive individuals responded to at least one SS peptide. 4/12 healthy volunteers responded to peptides of only one serotype, while the rest (8/12) responded to peptides of two DENV-serotypes.

Although, all the patients with acute infection had a secondary dengue infection, 16/26 patients did not have responses to any DENV SS specific peptides of any serotype suggesting altered DENV-specific T cell responses during acute infection. 6/10 patients who had DENV SS responses, responded to at least 1 SS peptide of DENV-1, 4/10 patients responded to at least 1 SS peptide of DENV-3, 5/10 responded to at least 1 SS peptide of DENV-2 and two patients responded to at least one SS peptide of DENV-4 (Table 2). 4/10 patients responded to SS peptides of only 1 serotype and 6/10 responded to SS peptides of 2 DENV serotypes (Table 2). Two of the patients who responded to SS peptides of 2 DENV serotypes, gave a history of a past symptomatic secondary dengue infection requiring hospitalization, which suggested that the current infection was third or fourth dengue infection. These two patients, who previously had a documented secondary dengue infection in the past, responded to SS peptide of DENV-3 and DENV-1 (CS10) and DENV-2/DENV-1 (CS31).

**Table 2:** Distribution of SS responses in those who responded to at least one SS peptide of the 4 dengue virus serotype in patients with acute infection.

	Clinical disease severity	DEN-1	DEN-2	DEN-3	DEN-4
CS32	DHF	A: D1 pep 1, D1 pep 11 C: same	A:D2 pep11, D2 pep 33 C: same	A: D3 pep 28 C: no response	
CS31	DHF	A: D1 pep 20	A: D2 pep 18		
CS10	DHF	A:D1 pep 11		A: D3 pep 21	
CS11	DF	A: D1 pep 20	A: D2 pep 1, D2 pep 33		
CS23	DF	A: D1 pep 11			
CS29	DF	A: D1 pep 20			
CS09	DF			A: D3 pep 21	
CS12	DF	A: D2 pep 1 C: no response		A: D3 pep 28 C: same	A: D4 pep 5 C: no response
CS15	DF		A: D2 pep 11, D2 pep 18		
CS16	DF		D2 pep 11		A: D4 pep 5

A indicates, responses to SS peptides during acute infection and C indicated responses to SS during the convalescent period.

The infecting DENV serotype could only be identified in 4/26 patients using PCR, and it was found to be DENV-1 which was compatible with the known dominant circulating dengue type in Sri Lanka [27]. Only one of these patients (CS29) responded to one of the SS peptides of the DENV-1 during acute infection. This patient (CS29) had DF. Four of the patients who did not respond to any SS peptide in the acute phase of the illness, responded to SS peptides in the convalescent phase of the illness. This further supported the possibility that dengue-specific immune responses were impaired during acute infection.

**Figure 1:**
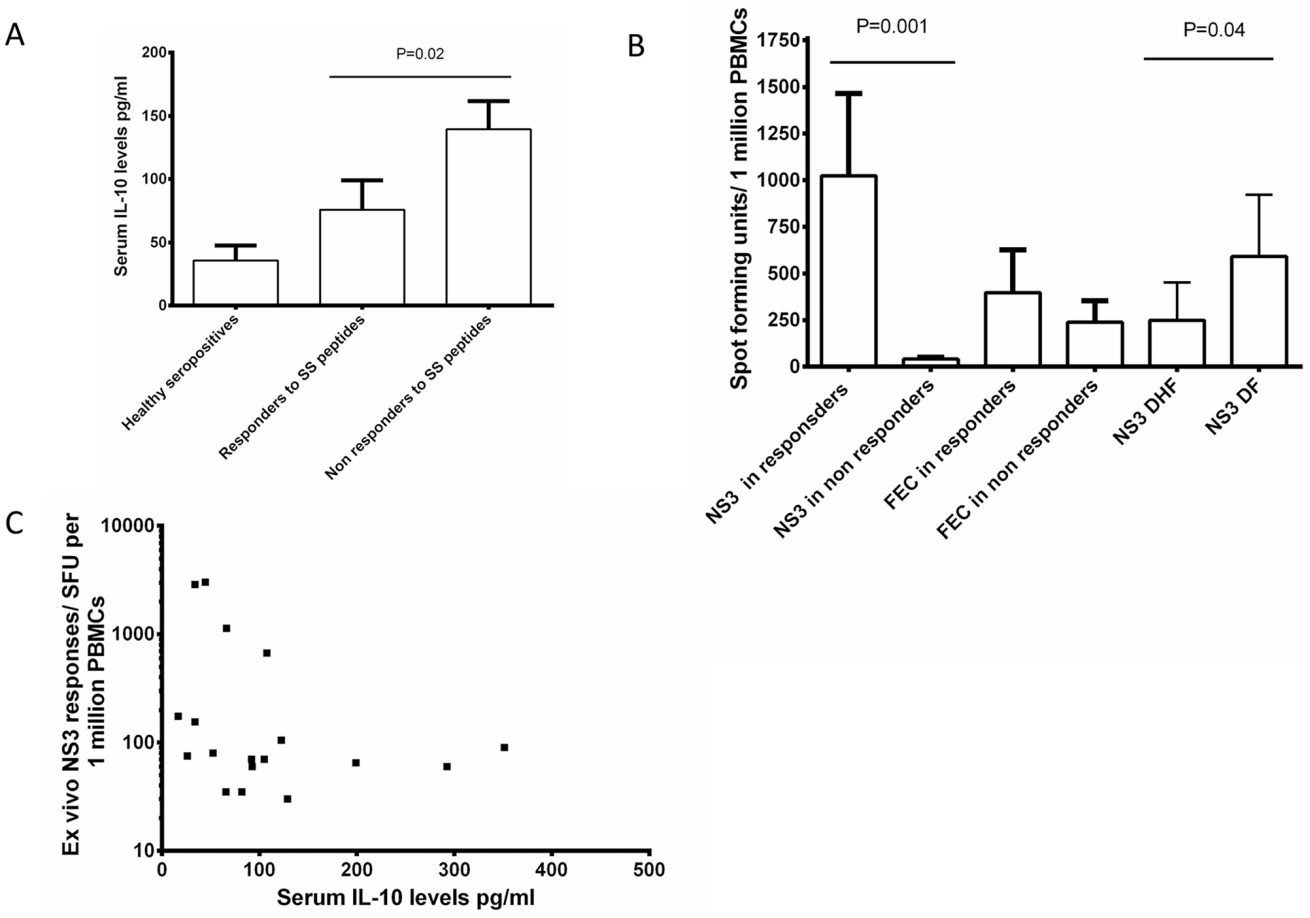
Serum IL-10 levels and DENV-specific immune responses. A: Serum IL-10 levels in healthy dengue seropositive individuals (n = 12), patients with acute dengue infection who did not respond to any of the SS peptides (n = 16) and patients who made responses to DENV-SS peptides (n = 10). The boxes represent the means and the horizontal bars the SEM. B: *Ex vivo* IFNγ ELISpot responses to DENV-NS3 overlapping peptides and FEC pool of peptides, in patients who did not respond to any of the SS peptides (n = 16) and patients who made responses to DENV-SS (10) peptides and in patients with DF (n = 10) and DHF (n = 16). The boxes represent the means and the horizontal bars the SEM. C: Correlation of *ex vivo* IFNγ ELISpot responses to DENV-NS3 overlapping peptides and serum IL-10 levels in patients with acute dengue infection (n = 23). P = 0.03 and Spearmans R = −0.45.

Although there was no statistically significant difference in the serum IL-10 levels in patients with DF and DHF (p = 0.28), the serum IL-10 levels were higher in patients with DHF (mean 140.2 pg/ml) when compared to patients with DF (mean 91.9 pg/ml). However, the serum IL-10 level were significantly higher (p = 0.02) in those who did not have DENV-SS specific responses (mean 144.2 pg/ml) when compared to those who responded (mean 75.7 pg/ml) (Fig. 1A). There were no differences in serum MIF levels or serum IL-21 levels in patients who responded to SS peptides and those who did not respond (data not shown). This raised the possibility that the impaired T cell responses could be related to IL-10 and was explored further below.

### DENV-NS3 and FEC specific T cell responses

DENV-NS3 specific IFNγ ELISpot responses were also significantly lower (p = 0.0001) in those who did not respond to DENV-SS peptides (mean 42 SFU/million PBMCs) when compared to those who responded SS peptides (mean 1024 SFU/million PBMCs) (Fig. 1B). However, there was no difference (p = 0.71) in IFNγ ELISpot responses to non-dengue FEC peptides in those with SS responses (mean 397.5 SFU/million PBMCs) when compared to those who did not have SS responses (mean 239.3 SFU/million PBMCs) (Fig. 1B). In addition, DENV-NS3 specific responses were significantly higher (p = 0.04) in patients with DF (mean 592.2 SFU/1 million PBMCs), than in patients with DHF (mean 249.3 SFU/1 million PBMCs). Serum IL-10 levels significantly (p = 0.03) and inversely (Spearmans R = −0.45) correlated with DENV-NS3 specific IFNγ ELISpot responses, but not with FEC responses (p = 0.52, Spearmans R = −0.14) (Fig. 1C). -

### Cytokine levels in ELISpot culture supernatants

**Figure 2:**
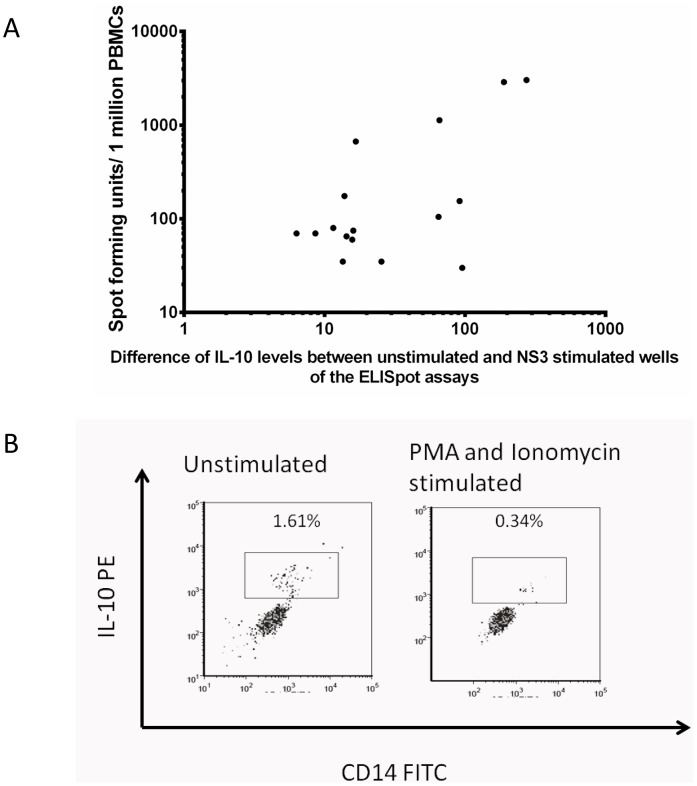
IL-10 production by PBMCs. A: Correlation of the difference of IL-10 levels (IL-10 levels in unstimulated wells minus IL-10 levels in NS3 stimulated wells) in ELISpot culture supernatants and *ex vivo* IFNγ ELISpot responses to DENV-NS3 overlapping peptides in patients with acute dengue infection. (P<0.0001 and Spearmans R = 0.81) B: Production of IL-10 by monocytes in patients with acute dengue infection. The cells are gated on CD3 APC negative and CD19 PerCP negative and CD14 FITC positive cells. Unstimulated (left panel) and PMA and Ionomycin stimulated (right panel) cells of a patient with acute dengue infection is shown.

We determined IL-10 and TGFβ levels in ELISpot culture supernatants in the unstimulated wells (media alone), NS3 stimulated and FEC stimulated wells. As described in our previous work [13], we again found that the IL-10 and the TGFβ levels in the unstimulated wells were higher than in the DENV-NS3 peptide stimulated wells and FEC stimulated wells. The difference in IL-10 levels in the DENV-NS3 peptide stimulated wells and the unstimulated wells were calculated by deducting the IL-10 concentrations in the unstimulated wells from the IL-10 concentrations in the NS3 stimulated wells. The difference of the IL-10 concentrations in the unstimulated and the NS3 stimulated wells (IL-10 values in unstimulated minus IL-10 values in NS3 stimulated wells) significantly (p<0.0001) and positively correlated (Spearmans R = 0.81), with *ex vivo* IFNγ ELISpot responses (Fig. 2A). No such association was seen in the difference between FEC stimulated wells and unstimulated wells (p = 0.09, Spearmans R = 0.36). This shows that the overall higher IFNγ ELISpot response correlates with lower secretion of IL-10 in stimulated wells. Although TGFβ levels were also higher in the unstimulated wells when compared to FEC and NS3 stimulated well, no associations were found with NS3 or FEC responses.

### Identification of the source of IL-10

Since IL-10 appeared to have a significant effect on DENV-specific T cell responses and also since PBMCs appeared to be producing significant amount of IL-10, we set out to identify the cells that were responsible for the production of IL-10 in the peripheral blood. For instance the mean IL-10 levels in unstimulated wells in the ELISpot plate, which only contained PBMC and media was 94.3 pg/ml. Using intracellular cytokine assays (ICS), we found that unstimulated PBMCs of patients with acute dengue infection produced a significant amount of IL-10 and the major source of IL-10 in the PBMC population was monocytes (Fig. 2B). Interestingly, during ICS, when PBMCs were stimulated with PMA and Ionomycin, the IL-10 production by monocytes drastically reduced (Fig. 2B) as observed in our ELISpot assays. ICS assays in B cells and T cells showed that insignificant amounts of IL-10 was produced from either T or B cells (<0.02% of T and B cells).

### Role of IL-10 in suppressing DENV-specific T cell responses

**Figure 3:**
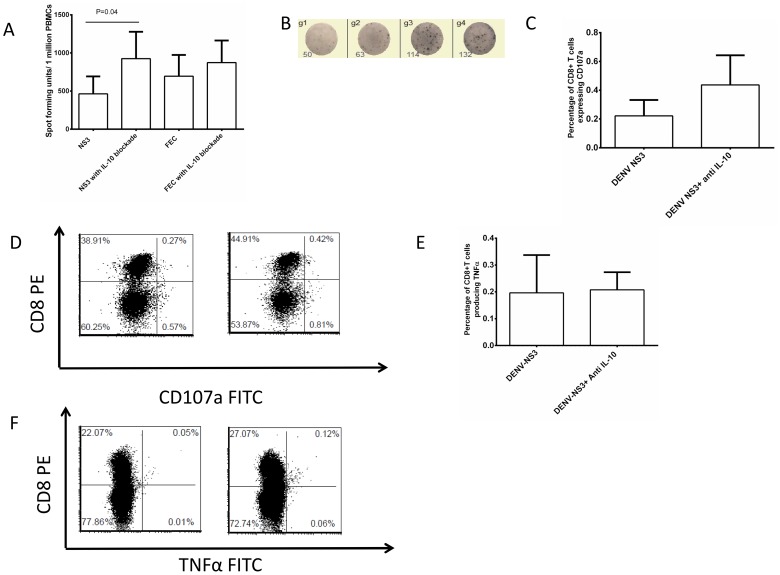
Effect of IL-10 blockade on antiviral T cell responses. A: *Ex vivo* IFNγ responses to DENV-NS3 overlapping peptides and FEC pool of peptides in patients with acute dengue infection, with and without IL-10 blockade (n = 10). The boxes represent the means and the horizontal bars the SEM. B: *Ex vivo* IFNγ responses to DENV-NS3 overlapping peptides in a patient with acute dengue viral infection, with and without IL-10 blockade. The left two wells are responses to NS3 without IL-10 blockade and the right two wells are responses to NS3 with IL-10 blockade. C: CD107a expression on CD8+ T cells in patients with acute dengue infection in the absence and the presence of IL-10 blockade (n = 6). The cells were gated on CD3+, CD8+ and Propidium Iodide negative cells. The PBMC were stimulated with DENV-NS3 overlapping peptides. The boxes represent the means and the horizontal bars the SEM. D. A dot plot of CD107a expression on CD3+ T cells in a patient with acute dengue infection. The cells were gated on CD3+ and Propidium Iodide negative cells. The PBMC were stimulated with DENV-NS3 overlapping peptide. The left dot-plot shows the responses toNS3 without IL-10 blockade and the right dot-plot is with IL-10 blockade. E: TNFα production by CD8+ T cells in patients with acute dengue infection in the absence and the presence of IL-10 blockade (n = 6). The cells were gated on CD3+ and CD8+ T cells. The PBMC were stimulated with DENV-NS3 overlapping peptides. The boxes represent the means and the horizontal bars the SEM. F: A dot plot of TNFα production in a patient with acute dengue infection. The cells were gated on CD3+ cells. The PBMC were stimulated with DENV-NS3 overlapping peptides. The left dot-plot shows the responses toNS3 without IL-10 blockade and the right dot-plot is with IL-10 blockade.

In order to determine if IL-10 could be suppressing DENV-specific T cell responses, we carried out *ex vivo* IFNγ ELISpots for NS3 and FEC in 10 of the patients with acute dengue and 4 healthy controls following blockade with anti-IL-10 and anti IL-10R antibodies. We found that IFNγ DENV-NS3 responses were significantly increased (p = 0.04) with IL-10 blockade (mean 925.4 SFU) when compared to no blockade (mean 463.2 SFU) (Fig. 3A and 3B). However, IL-10 blockade had no effect on FEC responses in patients with acute dengue infection (p = 0.11). IL-10 blockade also did not have any effect on DENV-NS3 specific responses in healthy dengue seropositive volunteers.

We then went on to determine if IL-10 had any effect on additional T cell effector functions such as TNFα production and degranulation (CD107a expression). Using ICS, with IL-10 blockade prior to adding the peptides, we found that although statistically not significant (p = 0.12) IL-10 blockade increased CD107a expression by DENV-NS3 specific T cells in 4/6 (Fig. 3C). The 2 patients did not have any CD107a expression when stimulated with DENV-NS3 and there was no response even after IL-10 blockade. Again although not statistically significant (p = 0.57) TNFα production by DENV-NS3 specific CD8+ T cells were also higher with IL-10 blockade (median 0.18 SEM±0.06) when compared to non blockade (median 0.025) (Fig. 3D).

### Effect of IL-10 blockade on Propidium Iodide expression on lymphocytes

As we used PI as a marker of dead cells in the ICS assays, we found that with IL-10 blockade, the cells appeared to be more viable in the forward and side scatter. Therefore, we went on to determine the effect of IL-10 blockade on lymphoid cells, CD3+ T cells and CD8+ T cells. We found that IL-10 blockade significantly reduced PI expression on CD3+T cells (p = 0.03). The PI mean expression on CD3+ T cells with IL-10 blockade was 36.6% whereas the mean expression on CD3+ T cells in the absence of IL-10 blockade was 42.7. PI expression was also reduced in CD8+ T cells with IL-10 blockade (mean 54.6%), when compared to those without IL-10 blockade (mean 58.9%) although this was not statistically significant (p = 0.12).

## Discussion

Patients with more severe clinical disease have been shown to have higher serum IL-10 levels [17], [18]. In our previous studies, which were done in a large cohort of patients with dengue infection, we found that higher serum levels of IL-10 were associated with higher T cell apoptosis [13]. Therefore, in this study we investigated the role of IL-10 in patients with acute dengue infection and its effect on DENV specific T cells. We found that serum IL-10 levels were significantly higher (P = 0.02) in patients who did not have SS peptide responses.

For detection of SS peptides, we used a panel of previously published SS peptides originating from highly conserved regions of the DENV, which included peptides specific for all for serotypes of the DENV [21]. All the healthy dengue seropositive volunteers who did not have a documented dengue viral infection in the past responded to the SS peptides of at least one serotype. All the patients with acute dengue suffered from a secondary dengue infection and they should have responded to the SS peptides of the previous infecting serotype. However, only 10/26 patients did so. Although only 3/16 patients with DHF responded to the SS peptides of at least one DENV serotype, 7/10 patients with DF responded, suggesting that patients with milder form of clinical disease are more likely to have responses to SS peptides during acute infection. Interestingly, 2 patients with previously documented DHF due to a secondary dengue infection responded to SS peptides, of two DENV serotypes.

We found that patients who did not respond to any of the SS peptides also had a lower frequency of DENV-NS3 specific T cells, but no difference in responses to non dengue viral peptides (FEC). Therefore, patients who did not respond to SS peptides of any of the DENV-serotypes also appear to have a lower frequency of other DENV-specific T cell responses but not T cell responses to other viral proteins. In addition, patients with more severe forms of disease (DHF) also had significantly lower DENV- NS3 specific T cell responses than patients with DF. Interestingly, as seen for DENV-SS responses, patients with higher serum IL-10 levels had a lower frequency of DENV-NS3 overlapping peptide specific T cell responses. However, there was no association between serum IL-10 levels and responses to non dengue viral proteins (FEC peptides). Therefore, it appears that IL-10 only had an effect on DENV specific immune responses in acute dengue viral infection and did not appear to have any effect on existing memory T cell responses to non dengue viral peptides. This was further confirmed by the fact that IL-10 blockade increased a number of assays of antiviral responses such as IFNγ production, CD107a expression and TNFα production to DENV-NS3 overlapping pool of peptides but not to the FEC pool of peptides. The mechanism underlying this selectivity is unclear.

We found that in experiments where PBMCs from patients with acute dengue infection were incubated with anti-IL-10 antibodies and anti-IL-10 receptor blocking antibodies *in vitro*, IL-10 blockade significantly reduced T cell death in the PBMCs of these patients. In our previous studies, we had found that serum IL-10 levels significantly correlated with T cell apoptosis, while inversely correlating with T cell numbers [13]. In this study we found that serum IL-10 levels inversely correlated with DENV-NS3 specific T cell responses but not with T cell responses to non dengue viral proteins. In addition, patients with lower IL-10 levels also were more likely to have DENV-SS T cell responses. Therefore, it is possible that IL-10 preferentially causes apoptosis of DENV-specific T cells or causes apoptosis of activated T cells. Therefore, IL-10 blockade would thus lead to recovery of DENV-NS3 specific responses and also DENV-SS T cell responses.

High serum IL-10 levels have been shown to be associated with a worse outcome in many viral infections including influenza and hepatitis B [19], [20], [28], [29], while in some viral infections such as in Japanese Encephalitis virus infection high IL-10 levels were shown to be associated with a favorable outcome [30]. In a murine model of West Nile virus, blockade of IL-10 signalling has been shown to increase production of antiviral cytokines and improve the disease outcome [29]. Il-10 has shown to have many immunomodulatory properties and has shown to inhibit antiviral T cell responses in other viral infections such as hepatitis B viral infection and HIV in vitro models [31], [32]. In chronic viral infections such as hepatitis B, elevation of IL-10 was found to coincide with disease flares and *in vitro* blockade of IL-10 was found to enhance polyfunctional antiviral responses in CD8+ T cells [32]. We too found that in acute dengue viral infection, the frequency of DENV-specific T cell responses were lower in patients with higher serum IL-10 levels and antiviral responses such as IFNγ production, CD107a expression and TNFα production were increased with *in vitro* IL-10 blockade. Therefore, it appears that IL-10 contributes to disease pathogenesis by inhibiting DENV-specific effector T cell responses.

Although, with our data it is evident that IL-10 does appear to reduce DENV-specific T cell responses, it is not clear if this is associated with a worse disease outcome or not. We found that both DENV-SS peptide responses and DENV- NS3 specific T cell responses were lower in patients with DHF when compared to patients with DF. However, our results are contradictory to a previous study which has shown that patients with severe clinical disease were more likely to have a higher frequency of DENV-NS3 specific T cells [33]. However, the timing of determining NS3 responses in this cohort of patients is not clear and it is possible that higher DENV-NS3 specific responses were observed by Duangchinda et al due to a later time point of sample collection [33]. Many studies have shown that T cells are highly cross reactive in acute dengue infection and it is speculated that such cross reactive T cells are likely to contribute to disease pathogenesis by production of inflammatory cytokines and suboptimal control of virus infection [7], [10], [34]. However, some have questioned the possible role of cross reactive T cells in pathogenesis of dengue, suggesting that cross reactive T cells are unlikely to be involved causing severe disease [11]. Another study carried out in a large cohort of children with acute dengue, which investigated the timing of the appearance of DENV-NS3 epitope specific T cells, fluid leakage and thrombocytopenia showed that DENV-NS3 specific T cells appeared only after the occurrence of fluid leakage and thrombocytopenia suggesting that they probably did not contribute much to disease pathogenesis. A more recent study by Weiskopf *et al* shows that healthy individuals with past dengue infection, living in a dengue endemic areas had a high magnitude and polyfunctional T cell responses to a large number of DENV specific CD8+ T cell epitopes. In fact the frequency of T cell responses restricted by HLA- alleles associated with more severe disease was less in this population and the responses were skewed towards more conserved epitopes [12]. Therefore, the data further strengthen the likelihood that DENV-specific T cell responses may actually be associated with reduced severity of dengue.

Patients with more severe forms of disease have been shown to have higher viral loads and prolonged viraemia [16]. Studies that were done to determine the kinetics of plasma viraemia have shown that primary infection with certain serotypes were associated with a higher viral load, which took longer to resolve [35]. Duyen *et al* also showed that those who had the highest plasma viraemia at day 3 were more likely to have lower platelet counts and more severe vascular leak [36]. Collectively, these studies suggest that a suboptimal immune response or an impaired T cell response may lead to severe disease possibly by the inability to eliminate the virus. Therefore, it appears that IL-10 contributes to disease pathogenesis by impairing T cell responses to the DENV.

We found that the main source of IL-10 in acute dengue viral infection in the blood is from monocytes. In our earlier studies we found that IL-10 levels in the unstimulated ELISpot culture supernatants were higher in patients with severe dengue when compared to those with non severe dengue [13]. Therefore, spontaneous IL-10 production by monocytes appears to be higher in patients with severe clinical disease. The DENV is known to preferentially infect monocytes directly and through antibody dependant enhancement [37], [38]. Although studies have not been carried out to determine if higher infection rates of monocytes in acute dengue viral infection, is associated with a higher production of IL-10, many *in vitro* studies have shown that higher infection rates in monocytes are associated with production of higher levels of proinflammatory cytokines [37]. Therefore, it is possible that patients with higher infection rates in monocytes induced higher production of IL-10, which in turn contributes to disease pathogenesis.

In summary, we have found that both DENV- SS specific T cell responses and DENV-NS3 specific responses were impaired in patients with higher serum IL-10 levels. Serum IL-10 levels did not appear to have any effect on non dengue viral proteins such as FEC. IL-10 blockade significantly increased IFNγ production, CD107a expression and TNFα production in response to DENV-NS3 overlapping pool of peptides but not to non dengue viral proteins. Therefore, our results suggest that IL-10 could be contributing to disease pathogenesis by inhibiting DENV-specific T cell responses.

## Supporting Information

Checklist S1 STROBE Checklist.
(DOCX)
